# Supplementary material for: Evaluation of Salt Stress-Induced Changes in Polyamine, Amino Acid, and Phytoalexin Profiles in Mature Fruits of Grapevine Cultivars Grown in Tunisian Oases
Source: Plants (Basel). 2023 Nov 30;12(23):4031. doi: 10.3390/plants12234031 (PMC10707986; doi:10.3390/plants12234031)
Supplement: Supplementary file 1 [file plants-12-04031-s001.zip › plants-2706030-supplementary.pdf]

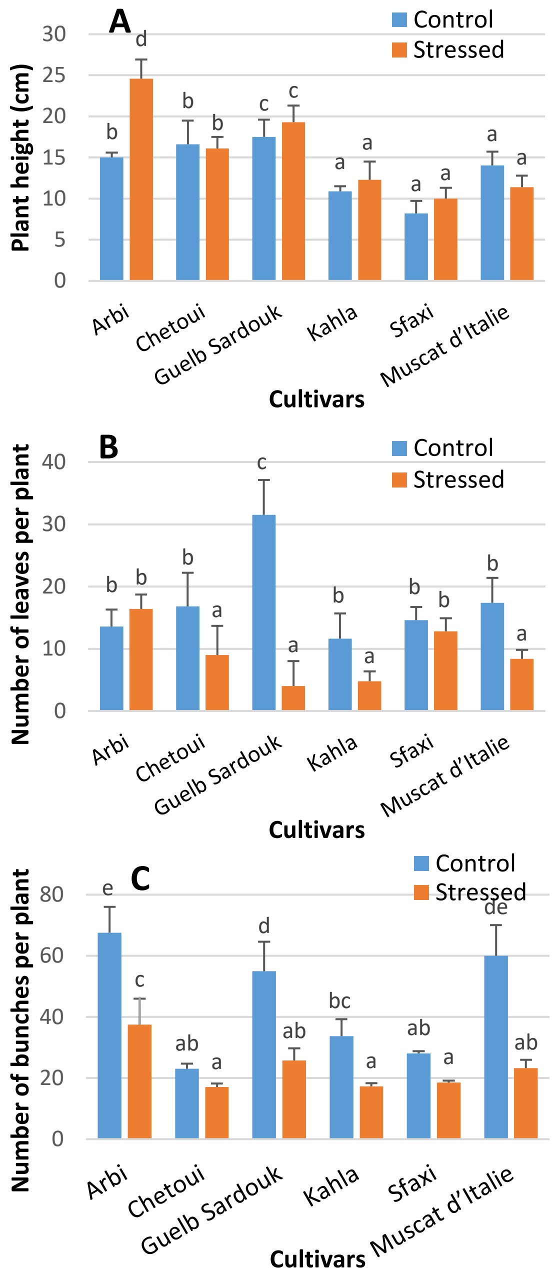

Supplementary Figure S1. Growth rate and yield of grape cultivars exposed to salt stress in the Oasis of El Jerid. Plant height (A), number of leaves per plant (B) and number of bunches per plant (C) in control and salt-stressed grapevine cultivars. Different letters indicate statistical significance between grape cultivars for each berry component, according to the Newman-Keuls test ($p < 0.05$).

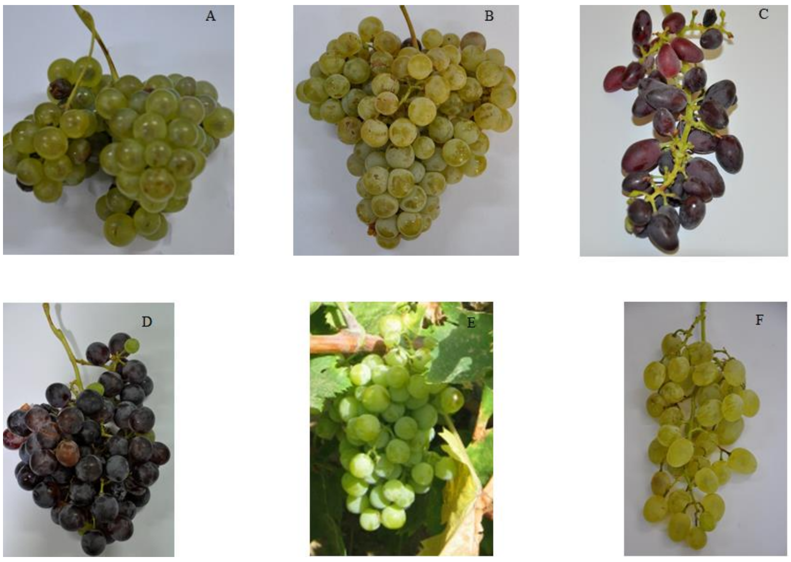

Supplementary Figure S2. Clusters of the studied grape cultivars at ripening. A: Arbi, B: Chetoui, C: Guelb Sardouk, D: Kahla, E: Sfaxi, F: Muscat d’Italie.

Supplementary Table S1. Ampelographic characteristics of studied grape cultivars in El Jerid Oasis.

Descriptor Code OIV / UPOV+	Descriptor	Arbi	Chetoui	Guelb Sardouk	Kahla	Sfaxi	Muscat d'Italie
	**Young shoot**						
[O-004,U-5]	**Density of prostrate hairs on tip**	Medium	Very dense	Absent	medium	Very low	Very high
[O-005,U-6]	**Density of erect hairs on tip**	Very sparse	sparse	Absent	Very sparse	Very low	Very sparse
	**Mature leaf**						
[O-065,U-19]	**Size of blade**	Medium	Medium	Small	Medium	Medium	large
[O-067,U-20]	**Shape of blade**	Wedge-shaped	Wedge-shaped	Wedge-shaped	Wedge-shaped	Wedge-shaped	Wedge-shaped
[O-084,U-33]	**Density of prostrate hairs between veins**	Absent	Very dense	Absent	Absent	Very low	Absent
[O-085,U-34]	**Density of erect hairs between veins**	Absent	Medium	Absent	Absent	Very low	Absent
[O-086,U-35]	**Density of prostrate hairs on main veins**	Very sparse	Very dense	Absent	Very sparse	Very low	Very sparse
[O-087,U-36]	**Density of erect hairs on main veins**	Very sparse	dense	Absent	Absent	Very low	Very sparse
	**Inflorescence and fruit**						
[O-204,U-40]	**Bunch : Density**	Loose	Loose	Very Loose	Loose	Loose	Loose
[O-223,U-43]	**Berry : Shape**	Round	Round	Arched	Round	Cylindric	Round
[O-225,U-44]	**Berry : Skin color (without bloom)**	Green-yellow	Green-yellow	Dark red-violet	Dark red-violet	Green-yellow	Green-yellow
[O-232,U-49]	**Berry : Juiciness of flesh**	Very juicy	Juicy	Slightly juicy	Juicy	-	Juicy
	**Seed**						
[O-243]	**100-seed weight**	Medium	High	High	High	-	High

Supplementary Table S2. Composition of irrigation water used in the oases of this study.

	**pH**	**ECe (dS m^{-1})**	**Salt load (g/L)**	**Temperature (C°)**	**Cl^- (mg/L)**	**Na^+ (mg/L)**	**K^+ (mg/L)**
**Oasis 1**	7,30	3,945	2,76	27,5	929	389,9	22,52
**Oasis 2**	7	8,02	5,61	27	2165	1345	66,6

Supplementary Table S3. Change of amino acid content (nmol.g^{-1} DW) in berry skin of grapevine cultivars exposed to high salinity. Different letters indicate statistical significance for each grape variety, according to Newman-Keuls test ($p < 0.05$).

	Arbi		Chétoui		Guelb Sardouk		Kahla		Sfaxi		Muscat d'Italie	
	Control	High salinity	Control	High salinity	Control	High salinity	Control	High salinity	Control	High salinity	Control	High salinity
Asn	45 ± 6 a	69 ± 3 b	36 ± 0 a	44 ± 1 b	86 ± 7 a	95 ± 3 a	35 ± 2 b	26 ± 1 a	59 ± 0 b	39 ± 2 a	89 ± 1 b	52 ± 1 a
**Ser**	208 ± 2 a	258 ± 16 a	149 ± 6 a	175 ± 8 a	357 ± 16 b	225 ± 7 a	158 ± 0 a	162 ± 2 a	265 ± 6 b	129 ± 4 a	428 ± 7 b	255 ± 8 a
Gln	52 ± 5 a	158 ± 9 b	242 ± 17 b	107 ± 17 a	360 ± 3 a	611 ± 3 b	103 ± 6 a	151 ± 10 b	181 ± 5 a	555 ± 27 b	423 ± 9 a	590 ± 12 b
**Arg**	1176 ± 48 a	2685 ± 125 b	2219 ± 38 b	437 ± 2 a	1442 ± 64 a	4542 ± 112 b	425 ± 7 a	630 ± 24 b	1190 ± 52 a	1918 ± 42 b	3585 ± 41 b	1310 ± 50 a
**Gly**	581 ± 22 a	988 ± 49 b	827 ± 25 b	389 ± 7 a	814 ± 47 a	1025 ± 55 b	248 ± 7 a	381 ± 12 b	653 ± 10 a	1035 ± 19 b	1095 ± 31 b	605 ± 0 a
Asp	196 ± 17 a	286 ± 12 b	307 ± 11 b	179 ± 14 a	400 ± 16 a	413 10 a	167 ± 14 b	109 ± 6 a	301 ± 1 a	303 ± 10 a	389 ± 1 b	250 ± 11 a
Glu	58 ± 5 a	93 ± 2 b	36 ± 2 a	117 ± 13 b	193 ± 11 a	203 ± 3 b	99 ± 4 b	76 ± 3 a	186 ± 2 b	73 ± 1 a	149 ± 6 a	145 ± 6 a
**Thr**	167 ± 13 a	192 ± 12 a	76 ± 3 a	128 ± 11 b	264 ± 9 b	157 ± 2 a	152 ± 5 b	78 ± 1 a	178 ± 13 b	100 ± 1 a	360 ± 2 b	205 ± 1 a
Ala	177 ± 55 a	345 ± 17 b	161 ± 7 a	167 ± 4 a	251 ± 13 b	150 ± 7 a	181 ± 5 b	145 ± 6 a	418 ± 5 b	222 ± 015 a	566 ± 8 b	408 ± 0 a
**GABA**	413 ± 3 a	493 ± 31 b	277 ± 19 a	339 ± 17 b	398 ± 7 b	171 ± 1 a	279 ± 6 a	269 ± 0 a	668 ± 22 b	124 ± 4 a	452 ± 12 a	444 ± 1 a
Cystin	8 ± 1 a	0,027 ± 1 b	10 ± 1 a	19 ± 1 b	14 ± 1 a	21 ± 0 b	4 ± 0 a	8 ± 0 b	18 ± 0 b	7 ± 0 a	21 ± 1 b	10 ± 1 a
**Pro**	976 ± 76 a	1045 ± 35 a	300 ± 4 a	5644 ± 147 b	652 ± 8 b	341 ± 3 a	479 ± 0 b	356 ± 15 a	637 ± 26 b	405 ± 10 a	1625 ± 39 b	408 ± 10 a
Orn	17 ± 1 a	87 ± 4 b	9 ± 1 b	3 ± 0 a	14 ± 1 a	33 ± 1 b	2 ± 0 a	4 ± 1 b	3 ± 0 a	17 ± 1 b	6 ± 0 a	24 ± 1 b
Cys	43 ± 5 a	210 ± 18 b	68 ± 9 b	24 ± 1 a	62 ± 0 a	267 ± 16 b	10 ± 0 a	38 ± 0 b	21 ± 1 a	108 ± 10 b	23 ± 2 a	88 ± 5 b
**Lys**	11 ± 0 a	50 ± 0 b	14 ± 0 b	4 ± 0 a	20 ± 0 a	29 ± 2 b	6 ± 0 b	4 ± 0 a	7 ± 0 a	13 ± 1 b	13 ± 0 a	10 ± 1 a
Tyr	297 ± 33 a	426 ± 13 b	146 ± 12 b	35 ± 1 a	134 ± 0 a	497 ± 18 b	43 ± 2 a	122 ± 4 b	33 ± 0 a	168 ± 1 b	225 ± 5 b	116 ± 9 a
**Met**	606 ± 51 b	546 ± 23 a	205 ± 23 a	679 ± 18 b	1451 ± 56 b	773 ± 10 a	719 ± 11 b	410 ± 12 a	883 ± 13 b	497 ± 20 a	1878 ± 87 b	586 ± 11 a
**Val**	2 ± 0 a	3 ± 0 b	1 ± 0 a	1 ± 0 a	2 ± 0 a	3 ± 0 b	1 ± 0 a	1 ± 0 a	1 ± 0 a	2 ± 0 a	2 ± 0 b	1 ± 0 a
**Ile**	26 ± 1 a	24 ± 1 a	9 ± 1 a	58 ± 1 b	85 ± 7 b	43 ± 0 a	46 ± 3 b	19 ± 1 a	31 ± 1 b	23 ± 1 a	76 ± 1 b	19 ± 1 a
**Leu**	80 ± 4 a	74 ± 3 a	22 ± 2 a	130 ± 10 b	223 ± 8 b	128 ± 12 a	114 ± 1 b	52 ± 1 a	101 ± 2 a	101 ± 6 a	220 ± 11 b	63 ± 3 a
**Phe**	23 ± 2 a	43 ± 3 b	11 ± 1 a	78 ± 4 b	32 ± 1 a	44 ± 2 b	26 ± 2 b	15 ± 0 a	28 ± 0 b	19 ± 1 a	19 ± 1 a	29 ± 1 b

Supplementary Table S4. Change of amino acid content (nmol.g^{-1} DW) in berry pulp of grapevine cultivars exposed to high salinity. Different letters indicate statistical significance for each grape variety, according to Newman-Keuls test ($p < 0.05$).

	Arbi		Chétoui		Guelb Sardouk		Kahla		Sfaxi		Muscat d'Italie	
	Control	High salinity	Control	High salinity	Control	High salinity	Control	High salinity	Control	High salinity	Control	High salinity
Asn	47 ± 0 a	654 ± 0 b	118 ± 9 b	35 ± 1 a	77 ± 2 a	226 ± 8 b	39 ± 0,000 a	39 ± 1 a	40 ± 1 a	62 ± 2 b	63 ± 1 a	90 ± 6 b
**Ser**	277 ± 30 a	376 ± 21 b	356 ± 13 b	217 ± 9 a	300 ± 6 a	390 ± 7 b	337 ± 29 a	407 ± 21 b	373 ± 9 a	390 ± 9 a	507 ± 26 a	573 ± 7 b
Gln	332 ± 44 a	664 ± 26 b	616 ± 2 b	165 ± 8 a	354 ± 18 a	1727 ± 56 b	211 ± 24 a	434 ± 7 b	497 ± 22 b	389 ± 13 a	869 ± 9 a	1939 ± 71 b
**Arg**	801 ± 72 a	4616 ± 399 b	1390 ± 45 b	237 ± 11 a	627 ± 34 a	4278 ± 156 b	470 ± 27 a	498 ± 17 a	551 ± 13 a	3407 ± 111 b	1859 ± 83 a	3002 ± 52 b
**Gly**	677 ± 54 a	1389 ± 42 b	842 ± 19 b	428 ± 30 a	763 ± 23 a	1072 ± 15 b	487 ± 4 a	658 ± 6 b	372 ± 27 a	1249 ± 23 b	979 ± 26 a	996 ± 6 a
Asp	132 ± 26 a	293 ± 53 b	230 ± 7 b	70 ± 8 a	143 ± 12 a	247 ± 6 b	77 ± 5 a	131 ± 6 b	184 ± 10 a	392 ± 19 b	182 ± 4 a	356 ± 1 b
Glu	188 ± 38 b	92 ± 6 a	46 ± 2 a	57 ± 3 b	49 ± 2 a	73 ± 5 b	82 ± 4 a	128 ± 15 b	83 ± 7 a	116 ± 0 b	105 ± 6 a	101 ± 6 a
**Thr**	142 ± 21 a	444 ± 27 b	124 ± 11 a	168 ± 4 b	235 ± 12 a	327 ± 16 b	232 ± 11 a	231 ± 12 a	217 ± 8 a	278 ± 15 b	330 ± 14 a	342 ± 4 a
Ala	363 ± 25 a	528 ± 75 b	180 ± 13 b	117 ± 11 a	245 ± 9 a	261 ± 25 a	439 ± 20 a	631 ± 56 b	402 ± 14 a	440 ± 15 a	564 ± 33 a	733 ± 3 b
**GABA**	344 ± 14 a	364 ± 53 a	192 ± 4 b	157 ± 2 a	125 ± 7 a	185 ± 10 b	556 ± 40 a	623 ± 19 b	481 ± 36 b	239 ± 6 a	306 ± 11 a	382 ± 15 b
Cystin	14 ± 1 a	23 ± 6 b	14 ± 1 a	13 ± 0 a	10 ± 1 a	26 ± 1 b	6 ± 0 a	19 ± 1 b	13 ± 1 a	13 ± 1 a	19 ± 0 a	24 ± 2 b
**Pro**	1582 ± 51 b	1041 ± 76 a	214 ± 8 a	8140 ± 323 b	593 ± 11 a	1387 ± 65 b	3013 ± 67 b	2064 ± 111 a	1310 ± 46 b	884 ± 42 a	1893 ± 7 b	1095 ± 65 a
Orn	4 ± 0 a	12 ± 1 b	5 ± 0 b	2 ± 0 a	5 ± 0 a	30 ± 7 b	3 ± 0 a	5 ± 1 b	2 ± 0 a	4 ± 0 b	5 ± 0 a	12 ± 0 b
Cys	64 ± 4 a	164 ± 3 b	113 ± 1 b	40 ± 4 a	55 ± 4 a	174 ± 10 b	50 ± 2 a	56 ± 2 a	68 ± 2 a	69 ± 4 a	90 ± 4 a	95 ± 2 a
**Lys**	11 ± 1 a	18 ± 0 b	14 ± 0 b	3 ± 0 a	5 ± 1 a	20 ± 3 b	4 ± 0 a	9 ± 0 b	6 ± 0 a	13 ± 0 b	7 ± 0 a	11 ± 0 b
Tyr	193 ± 12 a	464 ± 0 b	156 ± 6 b	116 ± 4 a	242 ± 9 a	997 ± 110 b	185 ± 6 a	197 ± 11 a	114 ± 2 a	245 ± 6 b	224 ± 5 a	306 ± 2 b
**Met**	481 ± 37 a	1374 ± 82 b	457 ± 6 a	421 ± 27 a	943 ± 11 a	1530 ± 60 b	1183 ± 71 a	1055 ± 42 a	812 ± 30 a	1253 ± 47 b	1286 ± 50 b	778 ± 14 a
**Val**	3 ± 0 a	6 ± 1 b	5 ± 0 b	3 ± 1 a	2 ± 0 a	4 ± 0 b	2 ± 0 a	2 ± 2 a	2,5 ± 0 a	3,5 ± 0 b	3 ± 0 a	3 ± 0 a
**Ile**	19 ± 0 a	146 ± 7 b	28 ± 1 b	22 ± 1 a	44 ± 5 a	93 ± 4 b	61 ± 1 b	30 ± 5 a	29 ± 1 a	56 ± 3 b	52 ± 2 b	36 ± 2 a
**Leu**	44 ± 2 a	147 ± 4 b	50 ± 1 b	35 ± 1 a	83 ± 7 a	163 ± 10 b	112 ± 3 b	87 ± 7 a	46 ± 1 a	119 ± 4 b	95 ± 6 b	61 ± 4 a
**Phe**	28 ± 4 a	353 ± 17 b	33 ± 2 a	53 ± 0 b	31 ± 2 a	154 ± 12 b	56 ± 5 a	56 ± 4 a	32 ± 1 a	46 ± 2 b	34 ± 1 a	80 ± 9 b

Supplementary Table S5. Change of amino acid content (nmol.g^{-1} DW) in berry seeds of grapevine cultivars exposed to high salinity. Different letters indicate statistical significance for each grape variety, according to Newman-Keuls test ($p < 0.05$).

	Arbi		Chétoui		Guelb Sardouk		Kahla		Sfaxi		Muscat d'Italie	
	Control	High salinity	Control	High salinity	Control	High salinity	Control	High salinity	Control	High salinity	Control	High salinity
Asn	364 ± 0 b	48 ± 4 a	25 ± 0 a	322 ± 8 b	151 ± 4 b	24 ± 2 a	107 ± 3 a	178 ± 4 b	148 ± 6 b	63 ± 2 a	56 ± 1 a	211 ± 4 b
**Ser**	104 ± 0 b	80 ± 2 a	95 ± 3 a	149 ± 4 b	68 ± 3 b	50 ± 0 a	71 ± 4 a	63 ± 2 a	37 ± 02 b	24 ± 1 a	18 ± 1 a	72 ± 5 b
Gln	213 ± 0 b	53 ± 4 a	70 ± 5 a	131 ± 5 b	75 ± 5 b	57 ± 2 a	75 ± 4 a	64 ± 4 a	38 ± 2 b	24 ± 1 a	30 ± 1 a	92 ± 9 b
**Arg**	557 ± 0 b	291 ± 28 a	321 ± 1 a	369 ± 4 b	233 ± 9 b	156 ± 2 a	276 ± 3 a	419 ± 14 b	140 ± 9 a	121 ± 5 a	118 ± 3 a	897 ± 63 b
**Gly**	504 ± 0 a	645 ± 0 b	214 ± 13 a	231 ± 8 a	314 ± 13 b	124 ± 6 a	271 ± 13 a	258 ± 13 a	141 ± 7 a	126 ± 9 a	121 ± 9 a	365 ± 20 b
Asp	148 ± 0 b	117 ± 5 a	69 ± 0,003 a	109 ± 5 b	72 ± 2 b	65 ± 1 a	73 ± 1 a	88 ± 3 b	98 ± 1 b	52 ± 4 a	47 ± 1 a	166 ± 8 b
Glu	456 ± 0 b	377 ± 6 a	344 ± 6 a	501 ± 3 b	352 ± 8 a	362 ± 9 a	297 ± 9 a	400 ± 2 b	652 ± 11 b	450 ± 24 a	325 ± 10 a	567 ± 19 b
**Thr**	53 ± 0 b	41 ± 1 a	2 ± 0 a	95 ± 8 b	40 ± 1 b	34 ± 1 a	41 ± 3 a	42 ± 1 a	35 ± 1 b	20 ± 1 a	15 ± 0 a	34 ± 1 b
Ala	205 ± 0 b	193 ± 0 a	4 ± 0 a	290 ± 10 b	95 ± 2 a	91 ± 1 a	118 ± 6 a	244 ± 17 b	103 ± 5 b	80 ± 6 a	57 ± 2 a	174 ± 7 b
**GABA**	199 ± 0 b	106 ± 8 a	153 ± 6 a	131 ± 9 a	90 ± 3 b	59 ± 1 a	141 ± 4 a	243 ± 8 b	76 ± 2 a	70 ± 1 a	77 ± 3 a	87 ± 4 a
Cystin	19 ± 0 b	11 ± 1 a	6 ± 0 a	15 ± 1 b	16 ± 0 b	6 ± 1 a	16 ± 1 a	16 ± 1 a	14 ± 0 a	16 ± 0 b	10 ± 1 a	24 ± 1 b
**Pro**	530 ± 0 b	266 ± 18 a	209 ± 12 a	541 ± 19 b	203 ± 3 a	256 ± 5 b	299 ± 8 b	272 ± 6 a	164 ± 9 a	276 ± 9 b	215 ± 10 a	209 ± 10 a
Orn	6 ± 0 b	4 ± 0 a	1 ± 0 a	12 ± 0 b	4 ± 0 b	1 ± 0 a	2 ± 0 a	3 ± 0 b	2 ± 0 a	4 ± 0 b	1 ± 0 a	5 ± 0 b
Cys	61 ± 0 b	29 ± 1 a	7 ± 0,001 a	51 ± 2 b	36 ± 5 b	9 ± 0 a	33 ± 2 b	23 ± 0 a	14 ± 0 a	45 ± 1 b	28 ± 1 a	41 ± 1 b
**Lys**	38 ± 0 b	20 ± 2 a	10 ± 0 a	37 ± 1 b	43 ± 2 b	18 ± 2 a	25 ± 2 a	27 ± 0 a	43 ± 0 b	29 ± 1 a	19 ± 0 a	32 ± 2 b
Tyr	208 ± 0 a	215 ± 1 a	136 ± 8 a	208 ± 5 b	180 ± 14 a	169 ± 22 a	218 ± 9 a	236 ± 11 a	215 ± 9 a	227 ± 10 a	123 ± 10 a	222 ± 10 b
**Met**	368 ± 0 a	381 ± 11 a	462 ± 4 a	773 ± 62 b	391 ± 18 b	254 ± 4 a	437 ± 5 b	319 ± 15 a	146 ± 1 b	103 ± 4 a	88 ± 7 a	185 ± 9 b
**Val**	6 ± 0 b	3 ± 0 a	2 ± 0 a	3 ± 0 b	4 ± 0 b	2 ± 0 a	4 ± 0 a	4 ± 0 a	5 ± 0 a	5 ± 0 a	3 ± 0 a	4 ± 0 b
**Ile**	24 ± 0 a	28 ± 0 a	34 ± 2 a	120 ± 10 b	66 ± 3 b	24 ± 0 a	29 ± 0 b	21 ± 1 a	12 ± 0 b	10 ± 0 a	3 ± 0 a	22 ± 1 b
**Leu**	129 ± 0 b	95 ± 2 a	87 ± 1 a	259 ± 16 b	148 ± 10 b	33 ± 2 a	65 ± 1 b	38 ± 2 a	18 ± 0 b	13 ± 0 a	12 ± 0 a	48 ± 0 b
**Phe**	95 ± 0 b	68 ± 5 a	26 ± 0 a	61 ± 3 b	64 ± 1 b	53 ± 1 a	77 ± 4 b	58 ± 1 a	102 ± 2 b	69 ± 0 a	66 ± 1 a	70 ± 1 b
